# Direct Engagement of TLR4 in Invariant NKT Cells Regulates Immune Diseases by Differential IL-4 and IFN-γ Production in Mice

**DOI:** 10.1371/journal.pone.0045348

**Published:** 2012-09-19

**Authors:** Ji Hyung Kim, Hye Sung Kim, Hye Young Kim, Sae Jin Oh, Doo Hyun Chung

**Affiliations:** 1 Department of Pathology, Seoul National University College of Medicine, Seoul, Korea; 2 Laboratory of Immune Regulation in Department of Biomedical Sciences, Seoul National University College of Medicine, Seoul, Korea; Tulane University, United States of America

## Abstract

During interaction with APCs, invariant (*i*) NKT cells are thought to be indirectly activated by TLR4-dependently activated APCs. However, whether TLR4 directly activates *i*NKT cells is unknown. Therefore, the expression and function of TLR4 in *i*NKT cells were investigated. Flow cytometric and confocal microscopic analysis revealed TLR4 expression on the surface and in the endosome of *i*NKT cells. Upon LPS stimulation, *i*NKT cells enhanced IFN-γ production, but reduced IL-4 production, in the presence of TCR signals, depending on TLR4, MyD88, TRIF, and the endosome. However, enhanced TLR4-mediated IFN-γ production by *i*NKT cells did not affect IL-12 production or CD1d expression by DCs. Adoptive transfer of WT, but not TLR4-deficient, *i*NKT cells promoted antibody-induced arthritis in CD1d^−/−^ mice, suggesting that endogenous TLR4 ligands modulate *i*NKT cell function in arthritis. Furthermore, LPS-pretreated WT, but not TLR4-deficient, *i*NKT cells suppressed pulmonary fibrosis, but worsened hypersensitivity pneumonitis more than untreated WT *i*NKT cells, indicating that exogenous TLR4 ligands regulate *i*NKT cell functions in pulmonary diseases. Taken together, we propose a novel direct activation pathway of *i*NKT cells in the presence of TCR signals via endogenous or exogenous ligand-mediated engagement of TLR4 in *i*NKT cells, which regulates immune diseases by altering IFN-γ and IL-4 production.

## Introduction

Invariant (*i)* natural killer T (NKT) cells express intermediate levels of a semi-invariant Vα24-Jα15 TCR in humans and Vα14-Jα18 TCR in mice [1], which recognize the glycolipid antigens presented by CD1d [2]. NKT cells regulate various immune responses in autoimmune diseases [3], [4], [5], tumor rejection [6], pulmonary fibrosis [7], and infection [8], [9]. Upon activation, *i*NKT cells rapidly produce large amounts of IL-4 and IFN-γ [10], which regulate innate and adaptive immune responses [11]. Therefore, it has been suggested that *i*NKT cells regulate immune diseases by modulating the Th1/Th2 balance *in vivo*.

Toll-like receptors (TLRs) are pathogen recognition molecules that activate the immune system as part of innate immunity [12]. TLRs expressed in dendritic cells (DCs) link innate and adaptive immunity by regulating the expression of chemokine receptors and adhesion molecules that allow DCs to mature and migrate into the draining lymph nodes [12]. TLR4 is complexed with MD-2 and CD14, and binds lipopolysaccharide (LPS) [13]. Upon ligand binding, TLR4 associates with myeloid differentiation factor 88 (MyD88) and Toll-interleukin-1 receptor domain-containing adaptor inducing IFN-β (TRIF), which is crucial for the recruitment of several proteins that are critical in signal transduction [14], [15]. MyD88-deficient mice have a profound defect in the activation of antigen-specific Th1 but not Th2 immune responses, suggesting that TLR signals play a critical role in balancing Th1/Th2 responses [16]. TLR4 specifically promotes production of the Th1-inducing cytokine IL-12 [17] and the chemokine interferon-gamma inducible protein (IP)-10 [18]. In contrast, Dabbagh and colleagues demonstrated that TLR4 is required for optimal Th2 responses against nonpathogenic antigens, which are dependent on DC maturation and cytokine production [19]. These conflicting data suggest that TLR4 plays a complex role in regulating the Th1/Th2 balance, depending on target tissue microenvironment. Upon TLR4 engagement, antigen presenting cells (APCs) are activated and produce IL-12 (cytokine-driven) and/or enhance presentation of endogenous glycolipid by CD1d molecules (self-antigen-driven) to *i*NKT cells, triggering their activation [20], [21]. The constitutive expression of high levels of IL-12 receptor endows *i*NKT cells with a rapid response to IL-12 and activation [22], [23]. Based on these findings, cytokine- and self-antigen-driven indirect pathways have been suggested to be the mechanisms by which TLR4-mediated activation of APCs regulates *i*NKT cell functions [21]. Therefore, it is generally accepted that *i*NKT cells are activated indirectly by TLR4-dependently activated DCs rather than directly by self-expressed TLR4 expressed.

Although the expression pattern and functions of TLR4 in *i*NKT cells remain controversial, TLR4 is expressed on the cell surface of naïve CD4^+^ T cells in mice and activated T cells in humans [24]. Moreover, TLR4 regulates subsequent TCR-dependent CD4^+^ T cell responses in experimental colitis in mice [25], [26]. These findings led us to hypothesize that engagement of TLR4 might directly activate *i*NKT cells, resulting in modulation of immune responses in various diseases. Therefore, to address this hypothesis, we investigated whether (i) TLR4 is expressed in *i*NKT cells and (ii) direct engagement of TLR4 modulates the function of *i*NKT cells *in vitro* and *in vivo*. Our data demonstrate that direct engagement of TLR4, expressed on the cell surface and localized in the early endosome, enhances IFN-γ production and reduces IL-4 production by *i*NKT cells, which modulates immune responses in various *i*NKT cell-mediated immune diseases.

## Results

### ***i***NKT Cells Constitutively Express TLR4 on the Surface and in the Early Endosome

**Figure 1:**
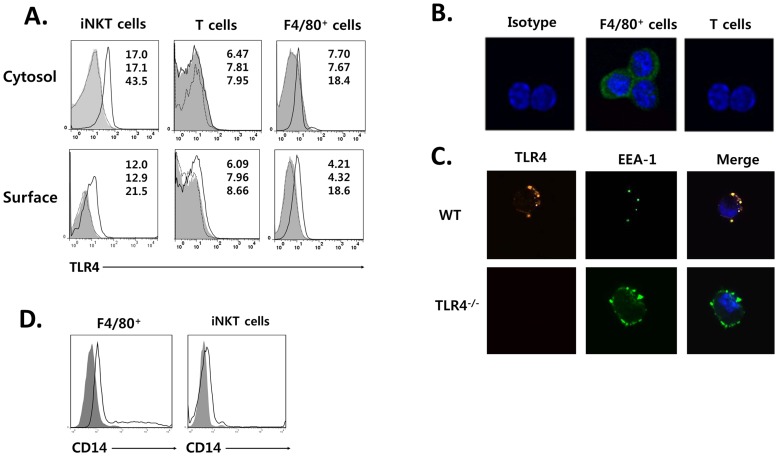
*i*NKT cells constitutively express TLR4 on the cell surface and in the endosomal compartment. (A) TLR4 expression was analyzed on gated α-GalCer/CD1d tetramer^-^CD3^+^ T cells, α-GalCer/CD1d tetramer^+^ *i*NKT cells, and F4/80^+^ macrophages from B6 (solid line) or TLR4^−/−^ mice (gray) compared with an isotype-matched control IgG (dotted line) by flow cytometric analysis. Numbers in diagrams represent mean fluorescence intensity (top for control, middle for TLR4^−/−^ mice, bottom for B6 mice). (B) Sorted *i*NKT cells and F4/80^+^ macrophages were stained with anti-TLR4 mAb (green) or isotype-matched control IgG, and DAPI (blue) (C) Sorted *i*NKT cells were stained with anti-TLR4 mAb or isotype-matched control IgG (red), and EEA-1 (early endosome marker; green) and DAPI (blue). (D) CD14 expression was analyzed on gated α-GalCer/CD1d tetramer^+^ *i*NKT cells and F4/80^+^ macrophages from B6 mice (solid line) as compared with an isotype-matched IgG control (gray). Data are representative of three independent experiments.

To explore whether *i*NKT cells express TLR4, liver mononuclear cells were obtained from wild type (WT) B6 or TLR4^−/−^ mice and TLR4 expression was examined both on the cell surface and in the cytoplasm of *i*NKT cells, macrophages, and conventional T cells. Flow cytometric and confocal microscopic analysis revealed that TLR4 was significantly expressed both on the cell surface and in the cytoplasm of macrophages and *i*NKT cells from WT, but not TLR4^−/−^ mice, whereas none of T cells from WT and TLR4^−/−^ mice expressed TLR4 (Fig. 1A–C). Moreover, TLR4 was co-localized with the early endosome maker EEA-1 in the cytoplasm of α-GalCer/CD1d tetramer^+^ *i*NKT cells from WT B6 mice, indicating that TLR4 is localized in the endosomal compartment of the cytoplasm in *i*NKT cells. Like as TLR4, both F4/80^+^ macrophages and *i*NKT cells also expressed CD14 on their surface (Fig. 1D). These findings indicate that α-GalCer/CD1d tetramer^+^ *i*NKT cells express TLR4 on the cell surface and in the cytoplasmic endosomal compartment.

### Direct Engagement of TLR4 in ***i***NKT Cells Enhances IFN-γ Production, but Suppresses IL-4 Production

**Figure 2:**
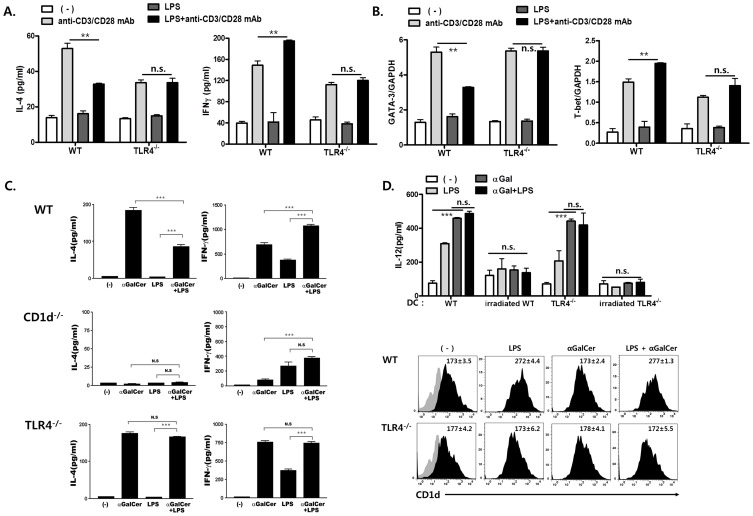
LPS-mediated direct engagement in *i*NKT cells enhances IFN-γ production, but reduces IL-4 production in the presence of TCR engagement. (A, B) Sorted *i*NKT cells from B6 or TLR4^−/−^ mice (1×10^5^/well) were stimulated using coated anti-CD3 (5 µg mL^−1^) + CD28 mAbs (5 µg mL^−1^) in culture plates, LPS (5 µg mL^−1^), or LPS (5 µg mL^−1^) + anti-CD3 (5 µg mL^−1^) + CD28 mAbs (5 µg mL^−1^) for 24 h. (A) The amounts of IL-4 and IFN-γ in the culture supernatant were measured by ELISA. (B) T-bet or GATA-3 mRNA expression were analyzed by real-time PCR. (C) B6, CD1d^−/−^, and TLR4^−/−^ mice were injected i.p. with α-GalCer (1 µg in 300 µl PBS), LPS (25 µg in 300 µl PBS), or α-GalCer (1 µg) + LPS (25 µg in 300 µl PBS). Serum IL-4 levels were monitored 2 h later, and serum IFN-γ levels were measured by ELISA 24 h after injection of these reagents. (D) *i*NKT cells were co-cultured with irradiated or un-irradiated bone marrow-derived DCs (BMDCs) from WT or TLR4^−/−^ mice in the presence of LPS and/or α-GalCer for 24 h. The levels of IL-12 in culture supernatant and CD1d expression on BMDCs were estimated. Numbers in diagrams represent mean fluorescence intensity. (A–D) Data are presented as the means ± SD of three mice in each group. Similar results were obtained from either two (D) or three (A–C) independent experiments. (*p<0.05, **p<0.01 and ***p<0.001).

To investigate the functional roles of TLR4 in *i*NKT and NK1.1^+^TCR-β^+^ NKT cells, the production of IL-4 and IFN-γ by sorted *i*NKT NK1.1^+^TCR-β^+^ NKT cells was measured after LPS and/or TCR stimulation. LPS-mediated TLR4 engagement reduced IL-4 production in B6 mouse *i*NKT and NK1.1^+^TCR-β^+^ NKT cells activated by anti-CD3+ CD28 mAbs, whereas LPS enhanced IFN-γ production (Fig. 2A and Fig. S1A, B). Moreover, LPS modulated IL-4 and IFN-γ production by activated *i*NKT cells in dose-dependent and TLR2-independent manners (Fig. S1C, D). Unlike WT NKT cells, LPS did not alter the production of these cytokines by TLR4-deficient *i*NKT and NK1.1^+^TCR-β^+^ NKT cells in the presence of anti-CD3+ CD28 mAbs (Fig. 2A and Fig. S1B). These results suggest that TLR4 engagement in TCR-mediated activated *i*NKT and NK1.1^+^TCR-β^+^ NKT cells enhanced IFN-γ production, but reduced IL-4 production.

T-bet and GATA-3 are key transcription factors that regulate the expression of Th1/Th2 cytokine genes, respectively [28], [29], [30]. Therefore, it has been hypothesized that T-bet and/or GATA-3 might contribute to the differential cytokine production by *i*NKT cells through TLR4 engagement. To address this hypothesis, we measured the transcriptional levels of GATA-3 and T-bet in gated *i*NKT cells from B6 or TLR4^−/−^ mice stimulated with anti-CD3+ CD28 mAbs and/or LPS (Fig. 2B). *i*NKT cells from B6 and TLR4^−/−^ mice stimulated with anti-CD3+ CD28 mAbs showed an increase in both GATA-3 and T-bet mRNA levels as compared with naive *i*NKT cells. In contrast, LPS-mediated TLR4 engagement reduced GATA-3 transcriptional levels and enhanced T-bet expression in WT *i*NKT cells, but not TLR4-deficient *i*NKT cells in the presence of TCR signals as compared with *i*NKT cells stimulated with anti-CD3+ CD28 mAbs alone. Although transcriptional levels of GATA-3 and T-bet do not completely reflect protein expression, these findings suggest that TLR4 engagement modulates the expression of GATA-3 and T-bet, which in turn differentially regulates Th1/Th2 cytokine production.

To confirm TLR4-mediated differential regulation of IL-4 and IFN-γ production by *i*NKT cells *in vivo*, we injected B6, CD1d^−/−^, or TLR4^−/−^ mice with α-GalCer and/or LPS and determined serum IFN-γ and IL-4 levels. α-GalCer treatment of B6 and TLR4^−/−^ mice significantly enhanced the production of IL-4 and IFN-γ, whereas CD1d^−/−^ mice showed no increases in the production of these cytokines (Fig. 2C). However, injection of α-GalCer and LPS into B6 mice reduced IL-4 production, but enhanced IFN-γ production in the sera, whereas the differential IL-4 and IFN-γ production by LPS and α-GalCer were not found in sera of TLR4^−/−^ mice. These results indicate that TLR4 engagement in *i*NKT cells enhances IFN-γ production and reduces IL-4 production in the presence of TCR stimulation *in vivo*. Collectively, TLR4 engagement in *i*NKT cells regulates T-bet and GATA3 expression, which in turn affects the production of IL-4 and IFN-γ. Next, to investigate whether this differential cytokine production affect DC function, IL-12 production and CD1d expression on DCs were measured during co-culture of *i*NKT cells and bone marrow-derived DCs (BMDCs) from WT or TLR4^−/−^ mice in the presence of LPS and/or α-GalCer. Treatment with α-GalCer + LPS did not alter IL-12 production in culture supernatant of *i*NKT cells and WT or TLR4-deficient BMDCs as compared with that of α-GalCer. However, IL-12 production in a culture containing irradiated BMDCs from WT or TLR4^−/−^ mice and *i*NKT cells was minimal. Furthermore, LPS and/or α-GalCer did not affect the expression of CD1d by TLR4-deficient BMDCs, whereas WT BMDC displayed enhancement of CD1d expression after LPS stimulation (Fig. 2D). These findings indicate that differential IFN-γ and IL-4 production by TLR4-dependently activated *i*NKT cells might not affect IL-12 production and CD1d expression by DCs during TLR4-mediated interaction.

### TLR4 Signals Mediated via MyD88 and TRIF Modulate Cytokine Production by ***i***NKT Cells, which is Dependent on Endocytosis and Endosome Formation

**Figure 3:**
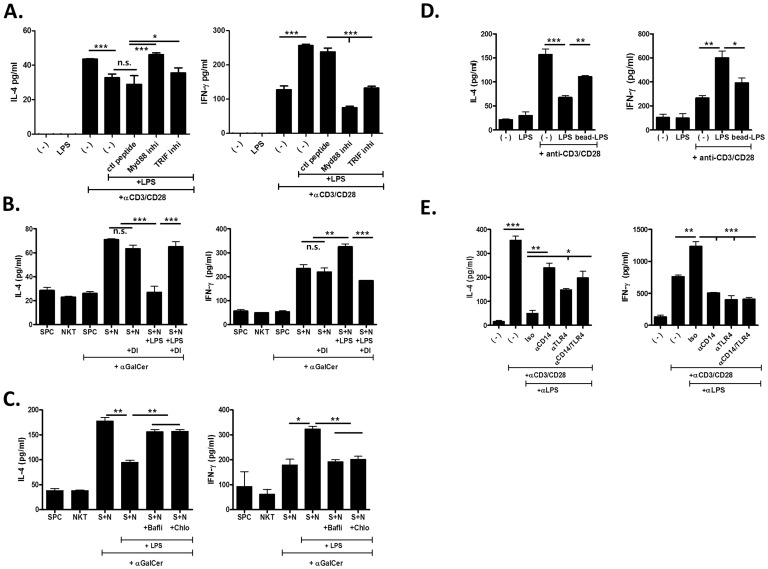
TLR4-mediated cytokine modulation by *i*NKT cells is triggered by MyD88 and TRIF, and depends on endocytosis and formation of the endosome compartment. (A, D, E) Sorted *i*NKT cells were incubated with anti-CD3+ CD28 mAbs or anti-CD3+ CD28 mAbs + LPS in RPMI media for 24 h. The concentration of IL-4 and IFN-γ was measured in culture supernatant by ELISA. (A) To block TLR4 signals, *i*NKT cells were pre-treated with Myd88 or TRIF inhibitor, or control (ctl) peptide for 1 h. *i*NKT cells (NKT or N), preincubated with 100 µM/ml of dynamin inhibitor (DI) (B), 100 nM/ml of bafiliomycin A1 (Bafli) for 30 min or 100 µM/ml of chloroquine (Chlo) for 2 h (C), were incubated with irradiated splenocytes (SPC or S) in the presence of α-GalCer. (D) *i*NKT cells were incubated with agarose bead-bound or unbound LPS in the presence of anti-CD3 and CD28 mAbs. (E) To inhibit surface TLR4 and/or CD14, *i*NKT cells were preincubated with anti-TLR4 mAb (25 µg/ml) or anti-CD14 mAb (50 µg/ml) for 30 min at 4°C before anti-CD3+ CD28 mAb stimulation. Data are representative of three independent observations. (n = 3 in each group; *p<0.05, **p<0.01, ***p<0.001).

Upon engagement of TLR4, activation signals are delivered into cells via the MyD88-dependent and TRIF-dependent pathways [15]. Therefore, to investigate whether TLR4-mediated signals in *i*NKT cells depend on MyD88 or TRIF, sorted *i*NKT cells were stimulated with LPS, anti-CD3+ CD28 mAbs, or LPS + anti-CD3+ CD28 mAbs in the presence of MyD88 or TRIF inhibitors, or a control peptide. MyD88 and TRIF inhibitors suppressed IFN-γ production, but enhanced IL-4 production by *i*NKT cells stimulated with anti-CD3+ CD28 mAbs and LPS as compared with the control peptide (Fig. 3A). These data suggest that signaling via TLR4 in *i*NKT cells depends on MyD88 and TRIF.

It has been reported that LPS triggers endocytosis of membrane TLR4 via the dynamin-dependent pathway, and TLR4 engages MyD88 and TRIF sequentially at the surface and in the endosome, respectively [31]. Therefore, to explore whether these events occur in TLR4 in NKT cells, *i*NKT cells were incubated with dynamin or endosome (Baflilomycin A [Balfi] and Chloroquine [Chlo]) inhibitors, and co-cultured with irradiated splenocytes of WT mice in the presence of LPS, α-GalCer, or LPS + α-GalCer. Treatment with dynamin or endosome inhibitors diminished LPS-induced cytokine modulation of IFN-γ and IL-4 by WT *i*NKT cells in the presence of TCR engagement (Fig. 3B, C). Furthermore, bead-bound LPS or blockade of surface TLR4 or CD14 using mAbs neither enhanced IFN-γ production and nor reduced IL-4 production by sorted *i*NKT cells in the presence of TCR signals as compared with unbound LPS or isotype-matched control mAbs, respectively (Fig. 3D, E). Consistent with these findings, CD14-independent (rough) LPS could not alter IL-4 and IFN-γ production by activated *i*NKT cells, suggesting that LPS-mediated effect on *i*NKT cells also depends on CD14 (Fig. S1E). These findings indicate that TLR4-mediated cytokine modulation by *i*NKT cells in the presence of TCR signals depends on endocytosis of LPS and TLR4, and early endosome formation.

### LPS-mediated Engagement of TLR4 in ***i***NKT Cells Aggravates *Saccharopolyspora Rectivirgula* (SR)-induced Hypersensitivity Pneumonitis, but Attenuates Bleomycin-induced Pulmonary Fibrosis

**Figure 4:**
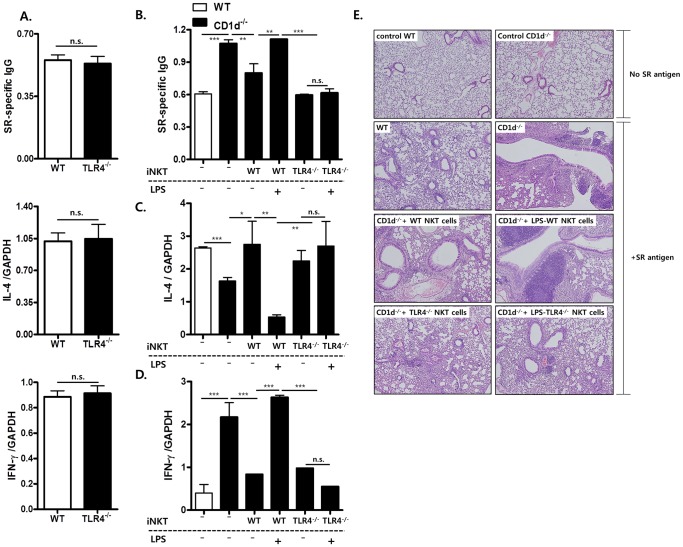
LPS-mediated engagement of TLR4 in *i*NKT cells aggravates *Saccharopolyspora rectivirgula* (SR)-induced hypersensitivity pneumonitis (HP). HP was induced by inoculating the SR antigen nasally. (A) The levels of SR-specific IgG in serum, and IL-4 and IFN-γ transcripts in the lungs of B6 or TLR4^−/−^ mice were analyzed seven days after the first nasal inoculation of SR antigen using ELISA and real-time PCR, respectively. (B–D) B6, CD1d^−/−^, and CD1d^−/−^ mice adoptively transferred with sorted *i*NKT cells (1×10^5^ cells) from WT B6 or TLR4^−/−^ mice were inoculated nasally with SR antigens. Sorted *i*NKT cells from B6 or TLR4^−/−^ mice were incubated with LPS or PBS for 30 min before adoptive transfer into CD1d^−/−^ mice. (B) These mice were sacrificed three weeks after induction of HP, and SR-specific IgG levels in serum were determined. (C) IL-4 and (D) IFN-γ levels were measured in the bronchoalveolar lavage fluid from B6, CD1d^−/−^, and CD1d^−/−^ mice adoptively transferred with sorted *i*NKT cells from B6 or TLR4^−/−^ mice seven days after inoculation of the first SR antigen by ELISA. (E) Histological examination of the lungs was performed 7 days after first SR treatment (×100). (A–D) Data are from a representative of three repeated experiments. (n = 4 in A, n = 3 in B, C, D; *p<0.05, **p<0.01, ***p<0.001).

**Figure 5:**
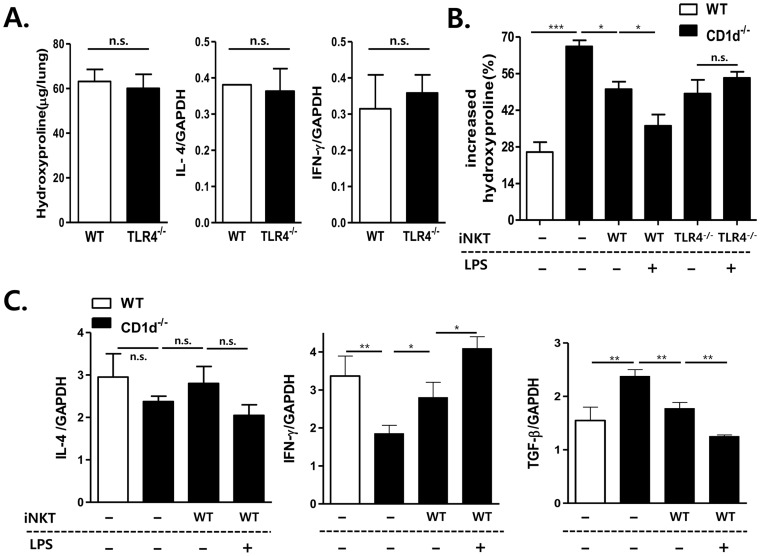
LPS-mediated engagement of TLR4 in *i*NKT cells suppresses bleomycin-induced pulmonary fibrosis. (A) Lungs were removed from B6 or TLR4^−/−^ mice 7 or 21 days after an intratracheal injection of bleomycin (2 mg/kg), and the levels of hydroxyproline, and IL-4 and IFN-γ transcripts were determined. (B) Hydroxyproline content in the lungs of B6, CD1d^−/−^, and CD1d^−/−^ mice adoptively transferred with sorted WT, TLR4-deficient *i*NKT cells, LPS-pretreated WT *i*NKT, or LPS-pretreated TLR4-deficient *i*NKT cells was determined 21 days after bleomycin injection. The increased hydroxyproline content in the lungs of experimental groups are expressed as a percentage. Data are indicated as mean ± SEM of six mice in each group. (C) The transcript levels of TGF-β1, IFN-γ, and IL-4 were determined by quantitative analysis relative to GAPDH using real-time PCR in the lungs of B6, CD1d^−/−^, and CD1d^−/−^ mice adoptively transferred with sorted WT *i*NKT or TLR4-deficient *i*NKT cells seven days after intratracheal injection of bleomycin. Data are representative of three repeated experiments. (n = 3 in each group; *p<0.05, **p<0.01, ***p<0.001).

Previously, we reported that IL-4-producing NKT cells play a protective role in SR-induced hypersensitivity pneumonitis (HP) by suppressing the IFN-γ-producing neutrophils, thereby decreasing SR-specific IgG levels, while NKT cells attenuate bleomycin-induced pulmonary fibrosis (BIPF) by producing IFN-γ [7], [32]. Therefore, based on TLR4-mediated differential IL-4 and IFN-γ production by NKT cells, we hypothesized that TLR4 engagement in *i*NKT cells reduces BIPF and aggravates SR-induced HP. To confirm this, we examined SR-induced HP and BIPF in WT and TLR4^−/−^ mice. However, there was no difference in the phenotypes of SR-induced HP and BIPF between WT and TLR4^−/−^ mice (Fig. 4A and 5A). Moreover, adoptive transfer of TLR4-deficient *i*NKT cells reduced both serum SR-specific IgG levels in HP and hydroxyproline levels in the lungs of CD1d^−/−^ mice in BIPF as much as did WT *i*NKT cells (Fig. 4B and 5B). Histological alteration in the lungs from mice with HP was consistent with serum SR-specific IgG levels, although histological alteration was not homogenous in the lungs with HP (Fig. 4E). These findings indicate that TLR4-deficient *i*NKT cells are functionally similar to WT *i*NKT cells in terms of their regulatory effects on HP and BIPF. These findings suggest that endogenous TLR4 ligands in *i*NKT cells are only minimally involved in the regulation of HP and BIPF. Therefore, to examine whether exogenous ligand LPS-mediated TLR4 engagement in *i*NKT cells modulates immune responses in SR-induced HP and BIPF, sorted WT or TLR4-deficient *i*NKT cells were pre-incubated with LPS *in vitro* and adoptively transferred into CD1d^−/−^ mice. LPS pretreatment of WT *i*NKT cells enhanced the levels of SR-specific IgG in serum and reduced hydroxyproline levels in the lungs of CD1d^−/−^ mice as compared with untreated WT *i*NKT cells (Fig. 4B and 5B). Real-time PCR revealed that adoptive transfer of LPS-pretreated WT *i*NKT cells increased IFN-γ production and reduced IL-4 production in the lungs of CD1d^−/−^ mice as compared with those mice adoptively transferred with untreated *i*NKT cells in the HP and BIPF models (Fig. 4C, D and 5C). In contrast, pretreatment of TLR4-deficient *i*NKT cells with LPS did not affect SR-specific IgG levels or hydroxyproline levels in the lungs in SR-induced HP and BIPF as compared with that of untreated TLR4-deficient *i*NKT cells. These findings suggest that exogenous but not endogenous TLR4 ligands affect *i*NKT cell function in SR-induced HP and BIPF.

### Endogenous Ligand-mediated TLR4 Signals in ***i***NKT Cells Promote Antibody-induced Arthritis

**Figure 6:**
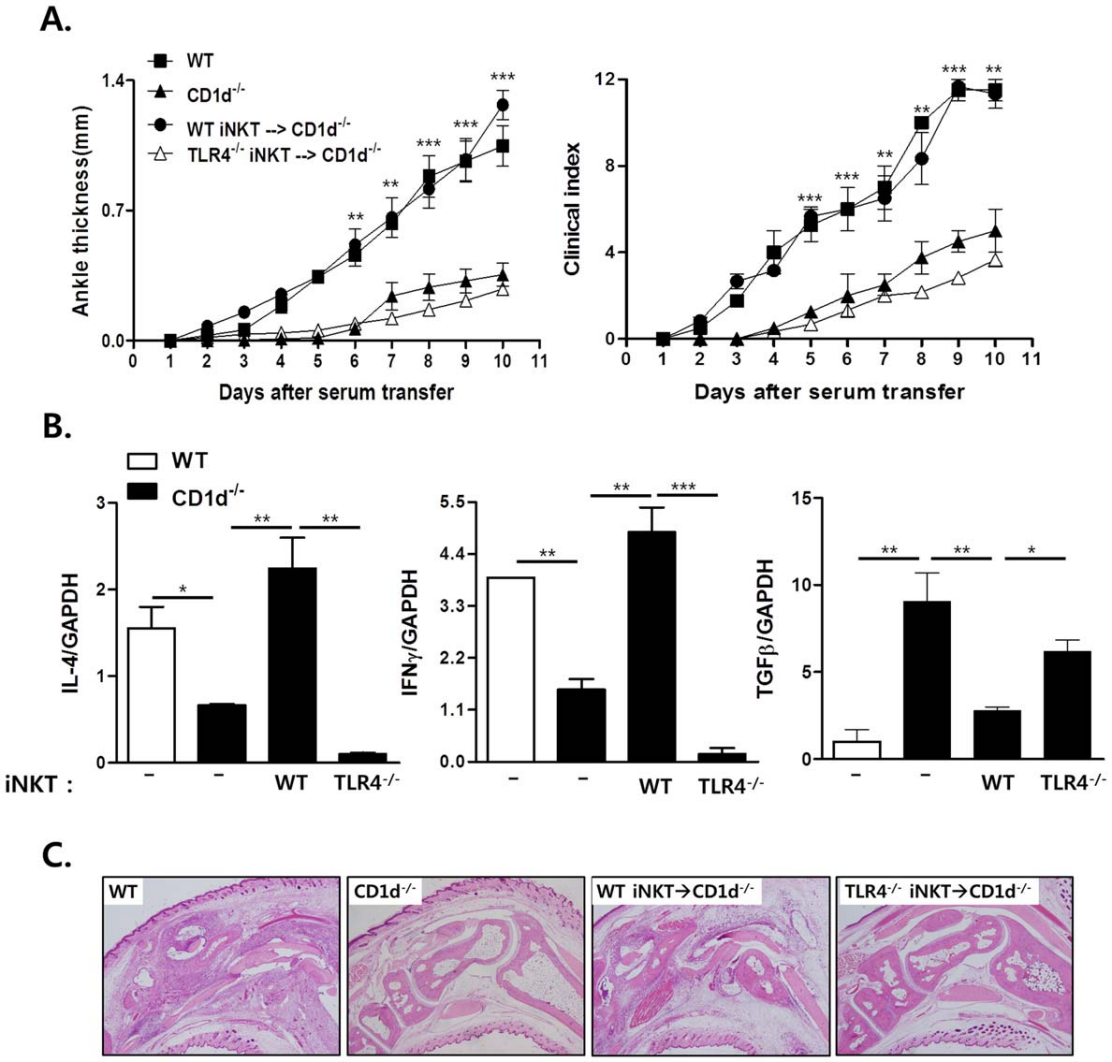
TLR4 in *i*NKT cells plays a crucial role in promoting antibody-induced joint inflammation. (A) Sorted WT or TLR4-deficient *i*NKT cells (3×10^5^cells mouse^−1^) were adoptively transferred into CD1d^−/−^ mice one day before K/BxN serum transfer (n = 3 per group). Clinical scores and ankle thickness were then monitored (*p<0.05 and **p<0.01, ***p<0.001). (B) The amounts of IL-4, IFN-γ and TGF-β1 were measured relative to GAPDH by real-time PCR in the joint tissues of B6, CD1d^−/−^, and CD1d^−/−^ mice administered sorted WT or TLR4-deficient *i*NKT cells 10 days after K/BxN serum transfer. (C) Histological examination of the joints was performed 7 days after K/BxN serum transfer (×100). Data are representative of three independent experiments. (n = 4 in each group; *p<0.05, **p<0.01, ***p<0.001).

To investigate whether TLR4-mediated differential production of IL-4 and IFN-γ by *i*NKT cells regulates *i*NKT cell-mediated immune diseases, we compared joint inflammation in CD1d^−/−^ mice adoptively transferred with either WT or TLR4-deficient *i*NKT cells in the K/BxN serum transfer model. In a previous report, we demonstrated that NKT cells promoted antibody-induced arthritis by producing IL-4 and IFN-γ [5]. WT *i*NKT cells promoted joint inflammation in CD1d^−/−^ mice as much as in B6 mice, whereas TLR4-deficient *i*NKT cells did not (Fig. 6A), which was consistent with histological alteration in the joints (Fig. 6C). Moreover, WT *i*NKT cells increased production of IL-4 and IFN-γ and suppressed TGF-β production in the joint tissues of CD1d^−/−^ mice, whereas TLR4-deficient *i*NKT cells did not alter cytokine production (Fig. 6B). To explain the failure of TLR4-deficient *i*NKT cells to promote arthritis, it is possible that TLR4-deficient *i*NKT cells might be functionally defective with respect to activation in the serum transfer arthritis model, thereby contributing to the failure to promote joint inflammation in CD1d^−/−^ mice. However, the ability of TLR4-deficient *i*NKT cells stimulated with α-GalCer to produce cytokines *in vitro* and *in vivo* indicates that TLR4-deficient *i*NKT cells are functionally intact in terms of activation by α-GalCer/CD1d complexes (Fig. 2A and C). Therefore, it is unlikely that the failure to promote antibody-induced joint inflammation in CD1d^−/−^ mice given TLR4-deficient *i*NKT cells was attributable to functional defects in TLR4-deficient *i*NKT cells. These data suggest that endogenous ligand-mediated signaling through TLR4 in *i*NKT cells contributes to their activation, resulting in the promotion of antibody-induced joint inflammation.

## Discussion

Data regarding the expression patterns of TLR4 in NKT cells are contradictory [33], [34], [35]. Nagarajan *et al.* suggested that neither TLR4 nor CD14 were expressed by *i*NKT cells [35]. In contrast, TLR4 expression was demonstrated in sorted NK1.1^+^TCR-β^+^ NKT cells by RT-PCR [33] and in the cytoplasm of *i*NKT cells using flow cytometry [34]. However, cell surface expression and cytosolic localization of TLR4 in *i*NKT cells was not fully characterized. Our experiments clearly demonstrate that *i*NKT cells expressed TLR4 on the cell surface and in the early endosome. Several lines of reasoning might explain the contradictory observations of TLR4 expression patterns in *i*NKT cells. TLR4 expression is constitutively detected in naïve murine T cells and down-regulated in activated murine T cells [25], [26], indicating that the level of TLR4 expression depends on activation status. However, constitutive expression of TLR4 in *i*NKT cells was not reduced after TCR-mediated activation (data not shown). Therefore, it is unlikely that the different activation status of *i*NKT cells in these experiments accounts for the contradictory expression pattern of TLR4 in *i*NKT cells. Moreover, it is also less likely that positive TLR4 expression pattern in NKT cells of our experiments might be attributable to contamination of other cell types in sorted NKT cells because purity of sorted NKT cells was extremely high (Fig. S1B). Alternatively, the sensitivity of the equipment, such as the flow cytometer, technical limitations, and/or the use of different reagents might affect detection of TLR4 expression by *i*NKT cells.

Although TLR4 has been reported to be expressed on the surface of most cell types [12], [13], [14], [16], it is also localized in the intracellular compartments of several types of cells including intestinal epithelial cells, placental syncytiotrophoblasts, dendritic cells, and neuroblastoma cells [36], [37], [38]. Moreover, LPS-induced TLR4 signaling in the cytoplasm of intestinal epithelial cells requires internalization of LPS [36], [39], [40]. Consistent with these findings, LPS triggers endocytosis of TLR4 on the surface, which is dependent on dynamin and early endosome formation. Furthermore, TLR4 sequentially engages MyD88 in the cell membrane and TRIF in the endosome [31]. In our experiments, TLR4-mediated differential IL-4 and IFN-γ production by *i*NKT cells was attenuated by treatment with anti-TLR4 or CD14 mAb, or inhibitors of dynamin, MyD88, TRIF, or the endosome. These data suggest that LPS binds TLR4 expressed on the cell surface and triggers endocytosis of TLR4, which in turn is re-localized to the early endosome, resulting in modulation of the functions of *i*NKT cells in MyD88- and TRIF-dependent manners.

Askenase *et al.* reported that stimulation of *i*NKT cells with LPS alone induced rapid, preferential, and transient production of IL-4, but not IFN-γ *in vitro* and *in vivo* [34]. In contrast, our experiments demonstrated that treatment of LPS alone did not alter production of IL-4 and IFN-γ by *i*NKT cells *in vitro*. Furthermore, LPS-mediated TLR4 signaling in *i*NKT cells enhanced IFN-γ production, but reduced IL-4 production in the presence of TCR signals. Consistent with our *in vitro* results, two independent studies demonstrated that stimulation of *i*NKT cells or CD1d-restricted T cell clones with LPS enhanced IFN-γ but not IL-4 production in the presence of DCs [41], [42]. Furthermore, in our *in vivo* experiments, LPS-mediated TLR4 signaling in *i*NKT cells enhanced IFN-γ production but reduced IL-4 levels in the lungs of mice with HP or BIPF, resulting in the regulation of these pulmonary diseases. Consistent with these results, *i*NKT cells have been reported to promote the development of LPS-induced lethal shock syndrome by producing IFN-γ [43]. Therefore, these findings suggest that signaling via TLR4 enhances IFN-γ production by *i*NKT cells in the presence of TCR engagement both *in vitro* and *in vivo*. Taken together, these data suggest that direct engagement of TLR4 in *i*NKT cells regulates immune responses by affecting the production of IFN-γ and IL-4 in the presence of TCR signaling.

In this study, TLR4 engagement directly activated *i*NKT cells, triggering differential production of IFN-γ and IL-4. Based on these findings, we suggest a novel TLR4-mediated direct activation pathway of *i*NKT cells during interactions between APCs and *i*NKT cells, in addition to the indirect pathways suggested by previous studies [20], [21]. However, it is unlikely that this differential cytokine production sequentially activates DCs to enhance IL-12 production and CD1d expression during interaction between DCs and *i*NKT cells. Therefore, it is conceivable that DAMPs (damage associated molecular pattern molecules) and PAMPs (pathogen-associated molecular patterns), released from damaged cells during tissue injury and inflammation, activate both DCs and *i*NKT cells by direct engagement of TLR4 expressed on these cells, and interactions between activated NKT cells and DCs further augments their activation status. Therefore, *i*NKT cells can be activated by either direct engagement of TLR4 and/or indirectly via IL-12 production (cytokine-driven) and enhanced presentation of endogenous glycolipid (self-antigen-driven) by TLR4-dependently activated DCs. This may contribute to the regulation of immune responses in various immune diseases including autoimmunity, transplantation, and tumor surveillance. Meanwhile, several groups have demonstrated that NKT cells directly recognize cell-wall glycosylceramides of the Gram-negative, LPS-negative *Sphingomonas* [44], [45], [46]. Therefore, in the case of bacterial infection, *i*NKT cells are activated not only via TLR4-mediated *i*NKT cell activation pathways that occur during tissue injury but also via direct recognition of bacterial glycolipids using the TCR.

With respect to the regulation of HP and BIPF, there was no functional difference between TLR4-deficient and WT *i*NKT cells in CD1d^−/−^ mice in adoptive transfer experiments. However, pretreatment of *i*NKT cells with LPS suppressed BIPF, but worsened SR-induced HP to a greater degree than did untreated-*i*NKT cells, indicating that exogenous rather than endogenous TLR4 ligands likely contribute to *i*NKT cell-mediated immune modulation in HP and BIPF. Unlike HP and BIPF, TLR4-deficient *i*NKT cells could not restore joint inflammation in CD1d^−/−^ mice in antibody-induced arthritis, whereas WT *i*NKT cells did. These findings suggest that endogenous ligands trigger TLR4-mediated signals in *i*NKT cells, resulting in the modulation of joint inflammation. Therefore, endogenous TLR4 ligand-mediated signals in *i*NKT cells promote inflammatory arthritis, whereas exogenous TLR4 ligands regulate *i*NKT cell functions in HP and BIPF. Consistent with these findings, endogenous ligand-mediated engagement of TLR4 is critical for the induction of rheumatoid arthritis [47], [48], [49], [50]. Taken together, exogenous and/or endogenous ligands directly engage TLR4 on *i*NKT cells *in vivo* and trigger TLR4-mediated immune modulation by *i*NKT cells, depending on the microenvironment of the disease model.

In conclusion, our experiments demonstrate that TLR4 is expressed on the surface and localized in the early endosome of *i*NKT cells. Direct engagement of TLR4 by either endogenous or exogenous ligands enhances IFN-γ production and reduces IL-4 production by *i*NKT cells. Moreover, TLR4-mediated differential cytokine production by *i*NKT cells modulates immune responses in various *i*NKT cell-mediated immune diseases such as SR-induced HP, BIPF, and antibody-induced joint inflammation in mice.

## Materials and Methods

### Mice

C57BL/6 mice were purchased from Orient Company (Seoul, Korea). CD1d^−/−^ mice were obtained from the NIAID-Taconic facility of the National Institutes of Health (Dr. Hua Gu, Columbia University, New York, NY). TLR4^−/−^ mice were a generous gift from Dr. Akira S (Osaka University, Osaka, Japan). KRN TCR transgenic mice and NOD mice were a gift from Drs. D. Mathis and C. Benoist (Harvard Medical School, Boston, MA) and from the Institute de Genetique et de Biologie Moleculaire et Cellulare (Strasbourg, France), and were maintained in a C57BL/6(B6) background (K/B). Arthritic mice (K/BxN) were produced by crossing K/B with NOD (N) mice. These mice were bred and maintained under specific pathogen-free conditions at the Clinical Research Institute Seoul National University Hospital, Seoul, Korea. All animal experiments were approved from the Institutional Animal Care and Use Committee at the Clinical Research Institute Seoul National University Hospital (IACUC No. 06212).

### Real-time PCR Analysis

To perform real time PCR, total RNA was isolated using an RNeasy kit (Qiagen, Courtaboeuf, France) according to the manufacturer’s instructions. RNA was reverse transcribed into cDNA using a MMLV-RT Taq polymerase (Promega, Madison, Korea) prior to PCR. For quantitative real-time PCR, gene-specific PCR products were measured using an Applied Biosystems 7500 Sequence Detection System (Perkin-Elmer Biosystem), and results for each cytokine were normalized versus GAPDH expression. The following primers and probe were synthesized by Applied Biosystems: GAPDH (TaqMan pre-developed Assay Reagent: 4352339E), IFN-γ (TaqMan pre-developed Assay Reagent: Mm 00801778 m1); TGF-β_1_, GCAACATGTGGAACTCTACCAGAA (forward), GACGTCAAAAGACAGCC ACTCA (reverse), and FAM-ACCTTGGTAACCGGCTGCTGACCC-TAMRA; IL-4 (TaqMan pre-developed Assay Reagent: Mm 00445259 m1); T-bet (TaqMan pre-developed Assay Reagent: Mm01299452_g1); and GATA-3 (TaqMan pre-developed Assay Reagent: Mm00484683_m1).

### Preparation of Mononuclear Cells from the Livers

To hepatic mononuclear cells, the livers were homogenized and resuspended in loading buffer (PBS plus 10% FBS and 1 mM EDTA) and overlaid onto lympholyte-M (Cedarlane, Ontario, Canada). After centrifugation for 20 min at 900×*g* at 15°C, hepatic mononuclear cells were isolated from the interface.

### Flow Cytometry

For flow cytometric analysis, mononuclear cells (1×10^6^) isolated from the liver were stained with antibodies for 30 min at 4°C. To perform intracellular staining, cells were surface stained, fixed, and permeabilized with Cytofix/Cytoperm according to the manufacturer’s instructions (BD Biosciences). PE-cy5-conjugated anti- mouse F4/80 (eBioscience, San Diego, CA, USA), APC-conjugated α-galactosylceramide (α-GalCer)/CD1d tetramer (NIH Tetramer Facility, NIH, Bethesda, MD), FITC-conjugated anti-mouse CD3e (BD Pharmingen, San Diego, CA, USA), FITC-conjugated anti-mouse CD14 (BD Pharmingen), and PE-conjugated anti-mouse CD1d (BD Pharmingen) antibodies were used.

### Confocal Microscopic Examination

Sorted *i*NKT cells from WT or TLR4-deficient mice were fixed with 4% formaldehyde for 30 min. After washing with phosphate buffered saline (PBS) for 5 min at 1500 rpm, cells were incubated with permeable/fixation solution (BD Pharmingen, San Diego, CA, USA) for 1 h at RT. After permeabilization, cells were incubated with APC-conjugated anti-mouse TLR4 (5 µg, eBioscience, San Diego, CA) and goat anti-mouse EEA-1 (1∶500; Santa Cruz, CA, USA) for 1 h at 30 min at RT, and then incubated with secondary antibody conjugated with Alexa 488 (10 µg; Molecular Probes, Eugene, OR). Slides were then stained with DAPI (4′.6-diamidino-2-phenylindole, 1∶2000, Invitrogen, Carlsbad, CA) for 5 min at RT and viewed at 100× magnification.

### Sorting and Activation of *i*NKT Cells

Liver α-GalCer/CD1d tetramer^+^CD3^+^ *i*NKT and NK1.1^+^TCR-β^+^ NKT cells were sorted using FACS Aria (BD Bioscience, San Diego, CA) and purity of sorted NKT cells was >99% (Figure S1A). Sorted *i*NKT or NK1.1^+^TCR-β^+^ NKT cells were incubated with αCD3+αCD28 mAb or αCD3+αCD28 mAb + LPS in RPMI media for 48 h. Twenty-four-well culture plates were coated with αCD3 mAbs in PBS (5 µg/ml) at 37°C for 2 h prior to incubation. To block TLR4 signaling, cells were pre-treated with Myd88 or TRIF inhibitors or control peptide (20 µM, Invivo Gen, San Diego, CA, USA) for 1 h and then washed with PBS. To inhibit surface TLR4 and/or CD14, sorted *i*NKT cells were preincubated with anti-TLR4 mAb (25 µg/ml) or anti-CD14 mAb (BD Pharmingen San Diego, CA, USA) (50 µg/ml) for 30 min at 4°C before stimulation with anti-CD3+anti-CD28 mAb.

### Inhibition of Endocytosis and Endosomal Compartments in *i*NKT Cells

The irradiated splenocytes (1000 rad) and *i*NKT cells were co-cultured with α-GalCer or α-GalCer+LPS for 24 h. Before co-culture, *i*NKT cells were preincubated with 100 µM/ml of dynamin inhibitor (Dynasore, Merck, Darmstadt, Germany), 100 nM/ml of bafilomycinA1 (Sigma, St. Louis, Mo., USA) for 30 min or 100 µM/ml of chloroquine (Sigma, St. Louis, Mo., USA) for 2 h. α-GalCer was synthesized using the method developed by Kim *et al.* [27].

### Reagents and Antibody

Smooth (CD14-dependent, catalog number; L8274) and rough (CD14-independent, catalog number; L9641) lipopolysaccharide (LPS; Sigma Chem. Co., St. Louis, MO) were used. Anti-TLR2 mAb was purchased from BD bioscience (San Diego, CA, USA), which neutralizes TLR2.

### Coating Agarose Beads with LPS

To inhibit internalization of LPS into the cells, LPS-coated beads were prepared using the AminoLink® plus Immobilization kit (Thermo Scientific Inc., Bremen, Germany) according to the manufacturer’s instructions. Briefly, LPS (2 mg) was diluted in 1 ml pH 7.2 coupling buffer, and end-over-end mixed with 4% beaded agarose supplied as 50% slurry in 0.02% sodium azide. Final LPS concentrations were determined using a Limulus ameboycte lysate (LAL) assay kit (Lonza, Walkersville, MD, USA).

### Preparation of Bone Marrow Dendritic Cells (BMDC) and Co-culture with *i*NKT Cells

To obtain BM cells, the tibia and femurs of WT and TLR4^−/−^ mice were flushed. CD4^+^, CD8^+^ T, B220^+^, and I-A^b+^ cells in BM were depleted using a magnetic bead separation kit (Miltenyi Biotec, GmbH, Bergisch-Gladbach, Germany). BM cells were incubated with 10 ng/ml of IL-4 (eBioscience, San Diego, CA, USA) and 20 ng/ml of GM-CSF (ProSpec-Tany TechnoGene, 51 Rehovot, Israel) for 5–8 d. BMDC differentiation was determined by flow cytometric analysis using a PE-conjugated anti-mouse CD11c mAb (>70% purity). Normal or irradiated BMDCs were co-cultured with *i*NKT cells stimulated with α-GalCer and/or LPS for 24 h.

### ELISA

For measuring cytokine production, sera and culture supernatants of sorted *i*NKT cells were collected and analyzed by ELISA. All cytokine ELISA kits were obtained from BD Biosciences, and ELISA was performed according to the manufacturer’s instructions. Standard curves were generated using known amounts of purified murine recombinant (r) IL-4, rIFN-γ, and rIL-12 (BD Biosciences, San Diego, CA, USA). The reaction was stopped with 3 N hydrochloric acid and the absorbance at 450 and 570 nm was read. Blood collected from the lateral tail veins was centrifuged, and SR-specific IgG levels in the serum (dilution 1/10) were determined by ELISA.

### Induction of hypersensitivity Pneumonitis (HP)

SR antigen was prepared from an SR strain obtained from the American Type Culture Collection (ATCC), and re-suspended in pyrogen-free saline. The SR antigen contained less than 20 ng/mg of endotoxin, estimated using a limulus amebocyte lysate assay (Sigma Chemical Co., St. Louis, MO). HP was induced by intranasally instilling 150 µg of SR antigen in PBS into mice under light anesthesia. This procedure was performed on three consecutive days per week for three weeks. Mice were sacrificed four days after the final treatment. Bronchoalveolar lavage (BAL) was performed by delivering 0.8 ml of ice-cold PBS into the cannulated trachea and gently aspirating the fluid.

### Induction of Bleomycin-induced Pulmonary Fibrosis (BIPF) and Hydroxyrproline Assays

Mice were anesthetized with 2, 2, 2-tribromomethanol, and intratracheally injected with 2 mg/kg of bleomycin (Nippon Kayaku, Tokyo, Japan) in PBS (50 µl). The total hydroxyproline level of the lung was determined as described previously [7].

### Serum Transfer and Arthritis Scoring

Arthritic adult K/BxN mice were bled and sera were pooled. Recipient mice were administered a 150 µl intraperitoneal injection of pooled K/BxN sera on days zero and two. Ankle thickness was measured with a caliper (Manostat, Switzerland). Joint swellings were monitored and scored as follows: 0, no joint swelling; 1, swelling of one finger joint; 2, mild swelling of wrist or ankle; 3, severe swelling of the wrist or ankle.

### Histological Examination

To evaluate histological alterations of the lungs and ankles, whole lungs or joint tissues were fixed in 10% formalin 7 days after first SR treatment or K/BxN serum transfer. Paraffin-embedded tissues were cut and stained with hematoxylin and eosin (H&E).

### Adoptive Transfer of *i*NKT Cells into CD1d^−/−^ Mice

Sorted *i*NKT cells (1×10^5^ cells per mouse) were adoptively transferred into CD1d^−/−^ mice by intravenous injection 1 day prior to the first intranasal instillation of SR antigen, bleomycin, or K/BxN serum transfer.

### Statistical Analysis

Statistical significance was analyzed using Prism ver. 5.0 (GraphPad Software, Inc., San Diego, CA, USA). A *t*-test was performed to compare two groups. To compare multiple groups, a one-way analysis of variance (ANOVA) using Turkey’s post hoc test was used. For all analyses, *P*<0.05 was considered statistically significant.

## Supporting Information

Figure S1 (A) Purity of sorted *i*NKT cells is >99% using FACS Aria. (B) LPS-mediated direct engagement in NK1.1^+^TCR-β^+^ NKT cells enhances IFN-γ production, but reduces IL-4 production in the presence of TCR engagement. Sorted NK1.1^+^TCR-β^+^ NKT cells from B6 or TLR4^−/−^ mice (1×10^5^/well) were stimulated using coated anti-CD3 (5 µg mL^−1^) + CD28 mAbs (5 µg mL^−1^) in culture plates, LPS (5 µg mL^−1^), or LPS (5 µg mL^−1^) + anti-CD3 (5 µg mL^−1^) + CD28 mAbs (5 µg mL^−1^) for 24 h. (C) Sorted *i*NKT cells were stimulated using coated anti-CD3 (5 µg mL^−1^) + CD28 mAbs (5 µg mL^−1^) in culture plates in the presence of various amount of LPS for 24 h. (D) Sorted *i*NKT cells were stimulated using coated anti-CD3 (5 µg mL^−1^) + CD28 mAbs (5 µg mL^−1^) in culture plates and LPS (5 µg mL^−1^) in the presence of isotype-matched control IgG or anti-TLR2 mAb (10 µg/ml) for 24 h. (E) Sorted *i*NKT cells were stimulated using coated anti-CD3 (5 µg mL^−1^) + CD28 mAbs (5 µg mL^−1^) in culture plates and CD14-independent LPS (5 µg mL^−1^) for 24 h. (B–E) The amounts of IL-4 and IFN-γ in the culture supernatant were measured by ELISA.
(TIF)
