# Supplementary figures and images for: Direct Engagement of TLR4 in Invariant NKT Cells Regulates Immune Diseases by Differential IL-4 and IFN-γ Production in Mice
Source: PLoS One. 2012 Sep 19;7(9):e45348. doi: 10.1371/journal.pone.0045348 (PMC3446883; doi:10.1371/journal.pone.0045348)

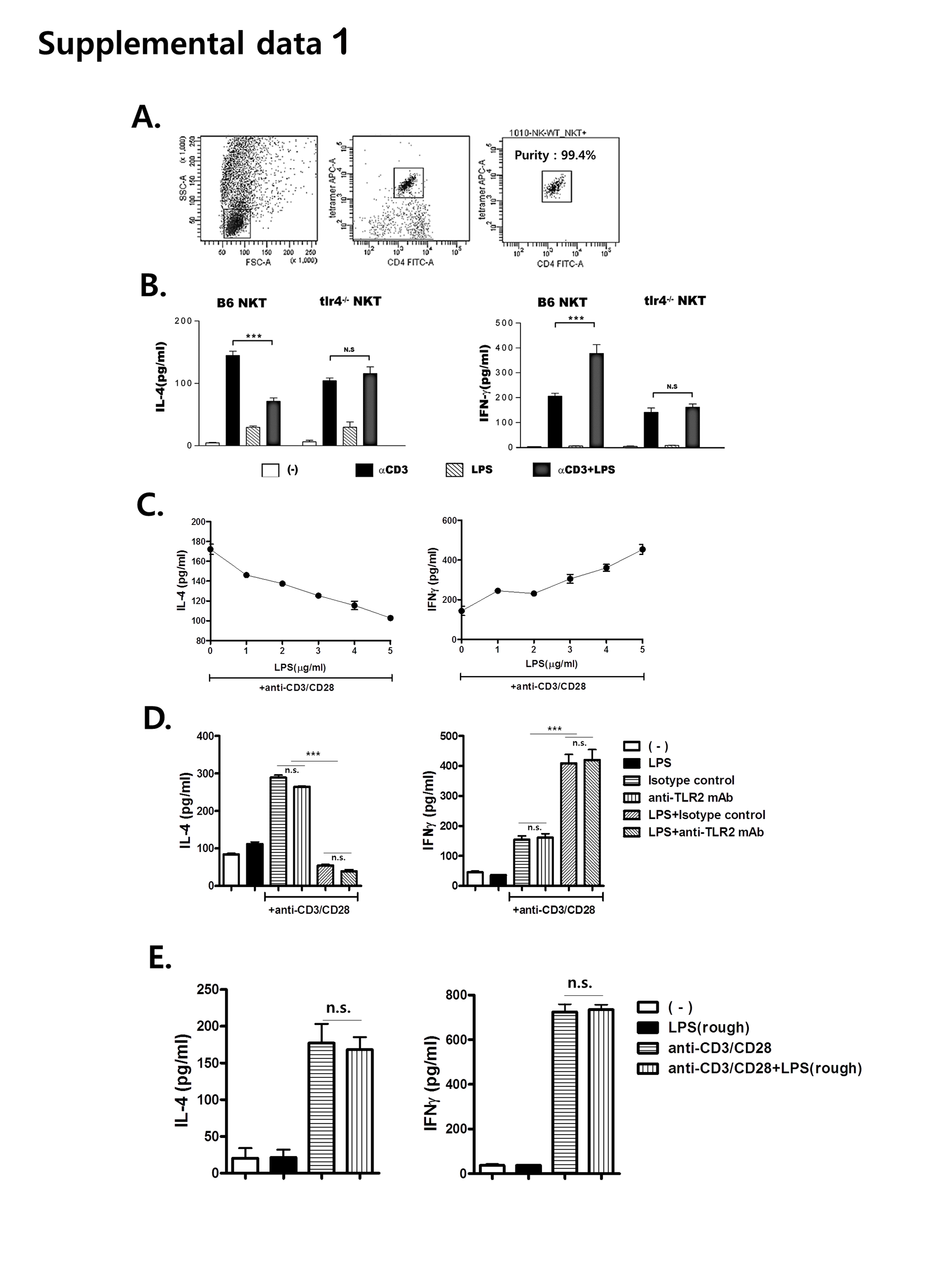

Supplement: Figure S1 — (A) Purity of sorted iNKT cells is >99% using FACS Aria. (B) LPS-mediated direct engagement in NK1.1+TCR-β+ NKT cells enhances IFN-γ production, but reduces IL-4 production in the presence of TCR engagement. Sorted NK1.1+TCR-β+ NKT cells from B6 or TLR4−/− mice (1×10^5^/well) were stimulated using coated anti-CD3 (5 µg mL−1) + CD28 mAbs (5 µg mL−1) in culture plates, LPS (5 µg mL−1), or LPS (5 µg mL−1) + anti-CD3 (5 µg mL−1) + CD28 mAbs (5 µg mL−1) for 24 h. (C) Sorted iNKT cells were stimulated using coated anti-CD3 (5 µg mL−1) + CD28 mAbs (5 µg mL−1) in culture plates in the presence of various amount of LPS for 24 h. (D) Sorted iNKT cells were stimulated using coated anti-CD3 (5 µg mL−1) + CD28 mAbs (5 µg mL−1) in culture plates and LPS (5 µg mL−1) in the presence of isotype-matched control IgG or anti-TLR2 mAb (10 µg/ml) for 24 h. (E) Sorted iNKT cells were stimulated using coated anti-CD3 (5 µg mL−1) + CD28 mAbs (5 µg mL−1) in culture plates and CD14-independent LPS (5 µg mL−1) for 24 h. (B–E) The amounts of IL-4 and IFN-γ in the culture supernatant were measured by ELISA. (TIF) [file pone.0045348.s001.tif]
